# Clinical Impact of Myocardium at Risk in Transcatheter Aortic Valve Implantation

**DOI:** 10.1161/CIRCINTERVENTIONS.125.015770

**Published:** 2026-05-20

**Authors:** Cristina Aurigemma, Giuliano Costa, Giulia Laterra, Thomas Pilgrim, Ignacio J. Amat Santos, Ole De Backer, Won-Keun Kim, Henrique Barbosa Ribeiro, Francesco Saia, Matjaz Bunc, Didier Tchetche, Philippe Garot, Flavio Luciano Ribichini, Darren Mylotte, Yusuke Watanabe, Francesco Bedogni, Tullio Tesorio, Tobias Rheude, Gennaro Sardella, Marco Tocci, Anna Franzone, Roberto Valvo, Sofia Sammartino, Mikko Savontaus, Hendrik Wienemann, Italo Porto, Caterina Gandolfo, Alessandro Iadanza, Markus Mach, Azeem Latib, Luigi Biasco, Maurizio Taramasso, Federico De Marco, Valentina Frittitta, Elena Dipietro, Claudia Reddavid, Orazio Strazzieri, Federica Agnello, Alessandro Comis, Mariachiara Calì, Mohamed Abdel-Wahab, Giulio Giuseppe Stefanini, Daijiro Tomii, Philippe Nuyens, Lars Sondergaard, Alessandro S Bortone, Marco Zimarino, Sergio F. Camara, Tullio Palmerini, Mateusz Orzalkiewicz, Klemen Steblovnik, Alexandre Gautier, Paolo Alberto Del Sole, Andrea Mainardi, Mattia Lunardi, Hideyuki Kawashima, Enrico Criscione, Vincenzo Cesario, Fausto Biancari, Federico Zanin, Giovanni Esposito, Matti Adam, Eberhard Grube, Stephan Baldus, Vincenzo De Marzo, Elisa Piredda, Stefano Cannata, Fortunato Iacovelli, Martin Andreas, Domenico Angellotti, Carmelo Sgroi, Erion Xhepa, Faraj Kargoli, Corrado Tamburino, Francesco Burzotta, Marco Barbanti

**Affiliations:** 1 Fondazione Policlinico Universitario A. Gemelli IRCCS, Roma, Italy (C.A., M.L., F. Burzotta).; 2 Division of Cardiology, A.O.U. Policlinico G. Rodolico-San Marco, Catania, Italy (G.C.).; 3 Università degli Studi di Enna Kore, Italy (G.L., M. Barbanti).; 4 Bern University Hospital, Inselspital, University of Bern, Switzerland (T. Pilgrim, D. Tomii).; 5 Centro de investigación Biomédica en red-Enfermedades Cardiovasculares (CIBERCV). Hospital Clínico Universitario de Valladolid, Spain (I.J.A.S.).; 6 Department of Cardiology, Algemeen Ziekenhuis Turnhout, Belgium (O.D.B., P.N.).; 7 Justus-Liebig University of Giessen, Cardiology & Angiology, Germany (W.-K.K.).; 8 Heart Institute of Sao Paulo (InCor), University of Sao Paulo, Brazil (H.B.R., S.F.C.).; 9 Cardiology Unit, Cardiac Thoracic and Vascular Department, IRCCS Azienda Ospedaliero-Universitaria di Bologna, Italy (F.S., T. Palmerini, M.O.).; 10 University Medical Centre Ljubljana, Slovenia (M. Bunc, K.S.).; 11 Clinique Pasteur, Toulouse, France (D. Tchetche, V.C.).; 12 Institut Cardio-vasculaire Paris Sud (ICPS), Hôpital Privé Jacques Cartier, Ramsay-Santé, Massy, France (P.G.).; 13 Division of Cardiology, Azienda Ospedaliera Universitaria Integrata di Verona, Italy (F.L.R., P.A.D.S., A.M.).; 14 Galway University Hospital, Ireland (D.M.).; 15 Department of Cardiology, Teikyo University School of Medicine, Tokyo, Japan (Y.W., H.K.).; 16 Division of Cardiology, IRCSS Policlinico San Donato, Italy (F. Bedogni, R.V., E.C.).; 17 Clinica Montevergine, GVM Care & Research, Mercogliano, Italy (T.T., F. Biancari, F.Z.).; 18 German Heart Centre, Munich, Germany (T.R., E.X.).; 19 Division of Cardiology, Policlinico Umberto I, Roma, Italy (G.S., M. Tocci).; 20 Department of Clinical Medicine, Copenhagen University, Denmark (O.D.B.).; 21 Division of Cardiology, AOU Federico II, Università di Napoli, Italy (A.F., G.E., D.A.).; 22 University of Catania, Italy (S.S., V.F., E.D., A.C., M.C.).; 23 Heart Center, Turku University Hospital, Finland (M.S.).; 24 Faculty of Medicine and University Hospital Cologne, Clinic III for Internal Medicine, Germany (H.W., M. Adam, E.G., S.B.).; 25 CardioThoracic and Vascular department, San Martino Policlinico Hospital, Genova, Italy (I.P., V.D.M., E.P.).; 26 Istituto Mediterraneo per i Trapianti e Terapie ad Alta Specializzazione (ISMETT), Palermo, Italy (C.G., S.C.).; 27 UOC Cardiologia Interventistica, Azienda Ospedaliera Universitaria, Siena, Italy (A.I.).; 28 Medical University of Vienna, Austria (M.M., M. Andreas).; 29 Montefiore Medical Center, New York, NY (A.L.).; 30 Azienda sanitaria locale di Ciriè, Chivasso e Ivrea, Italy (L.B., F.K.).; 31 HerzZentrum Hirslanden Zurich, Klinik Hirslanden, Switzerland (M. Taramasso).; 32 Centro Cardiologico Monzino, Milano, Italy (F.D.M.).; 33 Policlinico Umberto I, ASP Enna, Italy (C.R., O.S., F.A., M. Barbanti).; 34 Heart Center Leipzig at University of Leipzig, Germany (M.A.-W.).; 35 Department of Biomedical Sciences, Humanitas University, Milan, Italy (G.G.S.).; 36 IRCSS Humanitas Research Hospital, Milan, Italy (G.G.S.).; 37 Abbott Structural Heart, Santa Clara, CA (L.S.).; 38 Division of University Cardiology, Cardiothoracic Department, Policlinico University Hospital, Bari, Italy (A.S.B., F.I.).; 39 Department of Cardiology, SS. Annunziata Hospital Chieti, ASL 2 Abruzzo, Italy (M.Z.).; 40 Hôpital Paris Saint-Joseph, France (A.G.).; 41 ASP Catania, Italy (C.S.).; 42 Centro Cuore Morgagni, Pedara, Italy (C.T.).; 43 University of Nicosia, Cyprus (M. Taramasso).; 44 Università Cattolica del Sacro Cuore, Roma, Italy (F. Burzotta).; 45 Department of Neuroscience, Imaging and Clinical Sciences, G. d’Annunzio University of Chieti-Pescara, Italy (M.Z.).

**Keywords:** aortic valve stenosis, heart failure, humans, incidence, stroke

## Abstract

**BACKGROUND:**

The best management of coronary artery disease in patients with severe aortic stenosis undergoing transcatheter aortic valve implantation (TAVI) is debated. We investigated the clinical impact of the residual extent of myocardium at risk in patients undergoing TAVI.

**METHODS:**

Patients enrolled in the REVASC-TAVI (Management of Myocardial Revascularization in Patients Undergoing TAVI With Coronary Artery Disease) international multicenter registry were stratified according to the myocardium jeopardized by coronary artery disease using the British Cardiovascular Intervention Society Jeopardy Score (BCIS-JS) after a planned coronary revascularization. A planned revascularization included percutaneous coronary interventions performed before TAVI, during TAVI, or within 1 month after TAVI. The study population was divided according to the residual BCIS-JS (rBCIS-JS): patients with extensive residual myocardial at risk (rBCIS-JS >4 group) and patients without extensive residual myocardial at risk (rBCIS-JS ≤4 group). The primary study end point was the composite of all-cause death, nonfatal myocardial infarction, nonfatal stroke, and rehospitalization for heart failure at 2 years.

**RESULTS:**

Among the 2407 patients enrolled, 294 pairs of patients were selected by propensity matching and compared. At 2-year follow-up, the incidence of the primary end point was higher in patients with rBCIS-JS >4 compared with patients with rBCIS-JS ≤4 (37.5% versus 23.0%, *P*=0.004). A significantly lower rate of myocardial infarction was reported in patients with BCIS-JS ≤4 (8.2% versus 2.6%, *P*=0.011). At multivariate analysis, rBCIS-JS >4 (hazard ratio, 1.43 [95% CI, 1.11–1.84]; *P*=0.005) independently predicted 2-year major adverse cardiac and cerebrovascular events.

**CONCLUSIONS:**

In patients with concomitant coronary artery disease and severe aortic stenosis, the residual myocardial risk significantly affects TAVI outcomes. In particular, a rBCIS-JS >4 is associated with higher rates of major adverse cardiac and cerebrovascular events at 2 years.

**WHAT IS KNOWN**

- Coronary artery disease is common in patients undergoing transcatheter aortic valve implantation, but the best approach to revascularization in this setting remains unclear.
- Recent studies have yielded mixed results: 1 trial showed no benefit from routine pretranscatheter aortic valve implantation percutaneous coronary intervention, whereas another reported improved outcomes with percutaneous coronary intervention limited to significant lesions, leaving no consensus on managing coronary artery disease in transcatheter aortic valve implantation.

**WHAT THE STUDY ADDS**

- A residual British Cardiovascular Intervention Society Jeopardy Score above 4 was an independent predictor of adverse clinical outcomes at 2 years, underscoring the prognostic importance of residual myocardial ischemic burden in this population.
- These findings highlight the importance of a tailored coronary revascularization strategy before or around transcatheter aortic valve implantation to optimize prognosis.

Coronary artery disease (CAD) often coexists with degenerative aortic stenosis (AS), with over 50% of patients with AS undergoing surgical or transcatheter aortic valve implantations (TAVIs) also having CAD.^1–5^ In surgical cases, coronary artery bypass grafting is typically performed alongside valve replacement.^6,7^ However, for patients with TAVI, the optimal CAD management strategy remains unclear. Severe CAD correlates with higher mortality after TAVI, although performing percutaneous coronary intervention (PCI) in these patients can be challenging.^6–8^

Available retrospective data have reported conflicting results regarding the optimal approach to managing CAD in patients with TAVI.^5,8–13^ Two recent randomized clinical trials have significantly added to the current knowledge. On one hand, the ACTIVATION trial demonstrated that a routine revascularization strategy did not significantly reduce adverse outcomes after TAVI.^12^ On the other hand, the NOTION-3 trial demonstrated that, when guided by fractional flow reserve (FFR) assessment or an angiographic coronary artery diameter stenosis of at least 90%, PCI was associated with a lower risk of the composite outcome of death from any cause, myocardial infarction (MI), or urgent revascularization compared with conservative management, at 2 years after TAVI.^13^ Both trials excluded patients with CAD involving the left main.

Despite recent randomized evidence from the ACTIVATION and NOTION-3 trials, current recommendations remain imprecise and are largely supported by limited evidence, highlighting the persistent uncertainty regarding the optimal management of CAD in patients undergoing TAVI.^14^

In this context, the extent of jeopardized myocardium might constitute a possible modulator of CAD impact, but data in this field are lacking. Thus, we performed the present analysis from the multicenter REVASC-TAVI registry (Management of Myocardial Revascularization in Patients Undergoing TAVI With CAD) to investigate the clinical impact of the residual extent of myocardium at risk due to CAD in an all-comer TAVI population.

## Methods

The data that support the findings of this study are available from Prof Marco Barbanti (mbarbanti83@gmail.com) upon reasonable request.

### Data Source

Data for this analysis were obtained from the REVASC-TAVI data set. The REVASC-TAVI registry is an investigator-initiated study of patients with severe AS undergoing TAVI and found to have significant, untreated CAD at the time of pre-TAVI workup. The latter was defined as the presence of visual angiographic stenosis ≥70% (≥50% if protected left main or vein graft), instantaneous wave-free ratio value ≤0.89, FFR value ≤0.80, in ≥1 coronary arteries of at least 2.5 mm in diameter, not revascularized by patent coronary stents or bypass grafts, found at the coronary angiography performed during the pre-TAVI workup or left main minimal lumen area<6 mm^2^ at intravascular ultrasound assessment. The registry involves 30 centers across Europe, North America, South America, and Japan, with each center contributing patient-level data through a specialized case report form. The detailed protocols and methodology of the REVASC-TAVI registry have been previously published.^11^ Data on baseline demographics, clinical and echocardiographic features, medications, coronary angiography, PCI and TAVI procedural details and adverse events that occurred during the follow-up were collected into a dedicated case report form. The extension of coronary revascularization and the use of physiological assessment to guide the revascularization were selected at the operator’s discretion. The registry protocol received approval from the institutional ethics committee at each participating center. All patients involved in this study provided informed consent for the procedure.

### Study Population and Definitions

For the purpose of this analysis, patients who received planned PCI (before, during, or within 30 days after TAVI), with available follow-up and assessment of the extent of myocardial revascularization, were included. Those managed conservatively without any PCI were not part of this cohort. Jeopardized myocardium evaluated by the British Cardiovascular Intervention Society Jeopardy Score (BCIS-JS),^15^ was part of the original data set of the registry. PCI before TAVI was defined as PCI procedures planned and performed after the indication for TAVI and before the index TAVI procedure (PCI for acute coronary syndromes was excluded by definition). Concomitant PCI was defined as planned PCI procedures performed concomitantly during the index TAVI (either before or after transcatheter aortic valve deployment). PCI after TAVI was defined as PCI procedures planned and performed intentionally after TAVI in a different setting. Planned PCI included PCI performed either before TAVI, concomitantly or intentionally staged after TAVI. Unplanned PCI was defined as PCI procedures performed due to recurrent angina and acute coronary syndrome after TAVI.

**Figure 1.**
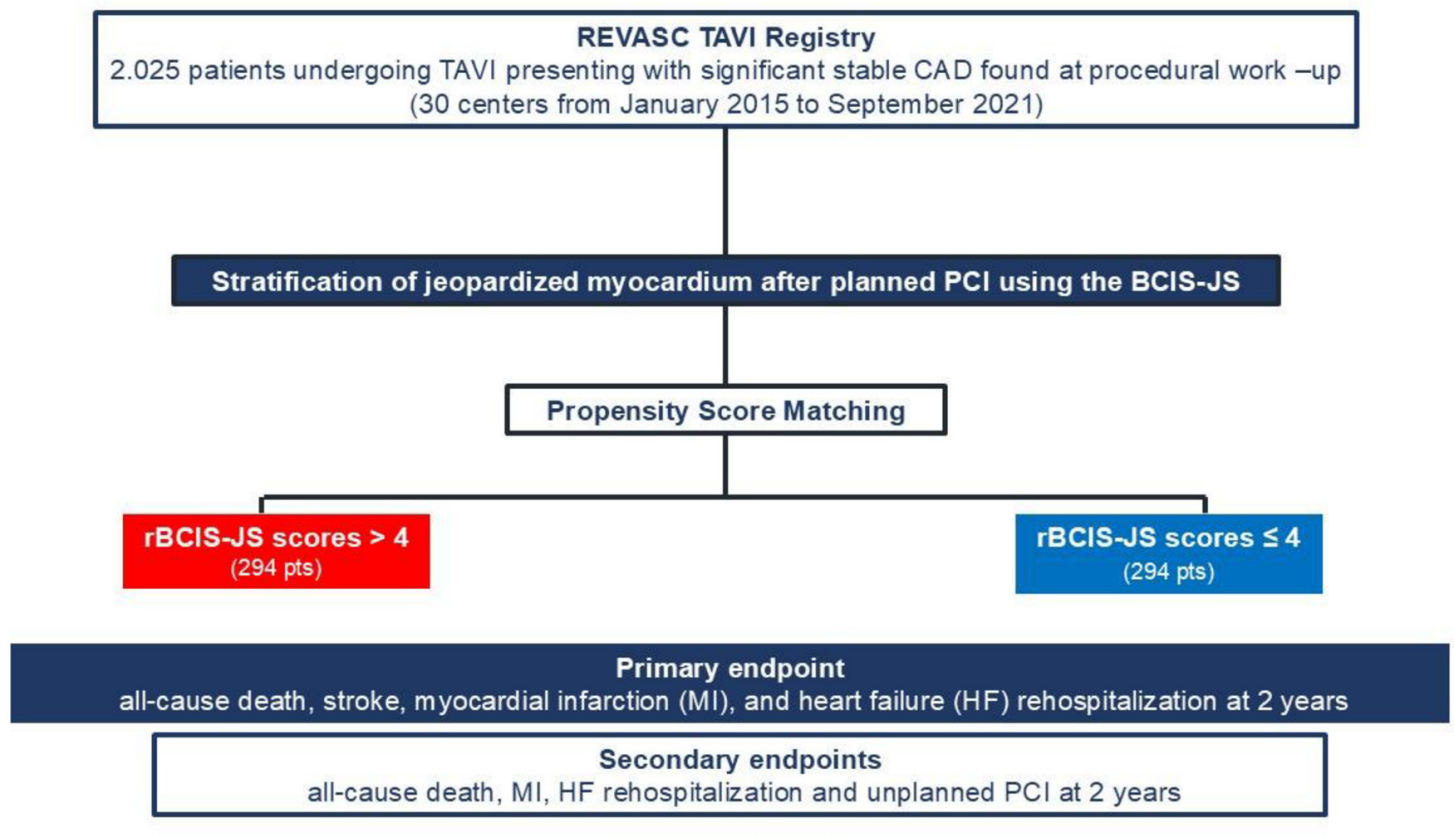
**Study flowchart.** The figure shows study design. BCIS-JS indicates British Cardiovascular Intervention Society Jeopardy Score; CAD, coronary artery disease; HF, heart failure; MI, myocardial infarction; PCI, percutaneous coronary intervention; rBCIS-JS, residual British Cardiovascular Intervention Society Jeopardy Score; REVASC-TAVI, Management of Myocardial Revascularization in Patients Undergoing Transcatheter Aortic Valve Implantation With Coronary Artery Disease; and TAVI, transcatheter aortic valve implantation.

For each patient with concomitant CAD, the jeopardized myocardium after planned PCI, was graded using the BCIS-JS.^15^ Briefly, the BCIS-JS quantifies the extent of myocardium at risk due to CAD. It assigns points to significant coronary stenoses (≥70% for major vessels and ≥50% for left main). The scoring prioritizes the major branch supplying the largest myocardial territory and accounts for anatomic variations. The final BCIS-JS ranges from 0 to 12, reflecting the myocardial area at risk from ischemia. A cutoff value of 4 for residual BCIS-JS (rBCIS-JS) has been validated in a different setting^10^ and was selected for the present study. Accordingly, the study population was stratified into 2 groups according to the rBCIS-JS: patients with extensive residual myocardial at risk (rBCIS-JS >4 group) and patients without extensive residual myocardial at risk (rBCIS-JS ≤4 group; Figure 1).

### Study End Points

The primary outcome was a composite end point including all-cause death, stroke, MI, and heart failure (HF) rehospitalization at 2 years. Secondary outcomes included MI, HF rehospitalization, and unplanned PCI at 2 years. All outcomes were defined according to the Valve Academic Research Consortium-2 definitions,^16^ as enrollment started before the publication of the Valve Academic Research Consortium-3 criteria. Clinical events defined according to Valve Academic Research Consortium-2 criteria were adjudicated locally by investigators at each center; no central event committee was used.

### Statistical Analysis

Categorical variables are reported as counts and percentages. Continuous variables are reported as medians and interquartile ranges. Continuous variables were compared with the *t* test or Mann-Whitney *U* test, and categorical variables were compared with the χ^2^ statistics, Fisher exact, or McNemar tests.

To account for baseline differences and selection bias, adjustment with propensity score matching (PSM) was used. Variables for the propensity score model were selected a priori based on clinical relevance and previous literature, adopting a nonparsimonious approach that included demographic, clinical, echocardiographic, and procedural characteristics potentially associated with outcomes in patients undergoing TAVI with concomitant CAD. Given the large sample size and number of events, the inclusion of multiple covariates was not expected to result in overfitting, as confirmed by standardized mean differences <10% after matching.

Variables included in the PSM were sex, age, body mass index, diabetes, hypertension, peripheral artery disease, chronic obstructive pulmonary disease, renal failure (defined as estimated glomerular filtration rate <30 mL/min), prior coronary artery bypass grafting, prior PCI, prior MI, prior stroke, prior pacemaker implantation, New York Heart Association classification, Canadian Cardiovascular Society classification, prior surgical aortic valve replacement, atrial fibrillation, Society of Thoracic Surgeons mortality score, left ventricular ejection fraction, aortic mean gradient, and aortic valve area (Figure S1). One-to-one PSM with the nearest neighbor method, with a caliper width of 0.1 of the SD of propensity score logit was used (Figure S1). A caliper of 0.10 was used for PS adjustment to achieve closer matching and minimize residual bias.

Time-to-event curves for the outcomes of interest were estimated using the Kaplan-Meier method. For secondary nonfatal end points, death represents a competing risk. As competing-risk methods were not applied, Kaplan-Meier estimates may overestimate the cumulative incidence of these events. Multivariable Cox-regression analysis was performed for investigating factors independently associated with the study outcome. Available clinical characteristics and the quantification of the residual myocardium at risk (by rBCIS-JS) were taken into account. Results were reported as hazard ratios (HRs) with 95% CIs. The proportional hazards assumption was assessed using Schoenfeld residuals, and no significant violations were observed.

All statistical tests were performed 2-tailed, and *P*<0.05 was considered as the threshold for statistical significance. All statistical analyses were performed with R software, version 3.6.3 (R Foundation for Statistical Computing).

## Results

The REVASC-TAVI registry enrolled 2404 patients with significant, stable CAD who underwent TAVI between January 2015 and September 2021. After excluding 377 patients due to a lack of follow-up data or extent of myocardial revascularization information, 2025 patients constituted the prematching cohort. No imputation of missing values was performed. Descriptive analyses indicated that excluded patients were comparable to included ones in terms of age and sex, and missingness appeared largely random across centers. Among patients considered for the present analysis, 408 (20.1%) received a conservative treatment for CAD. The baseline demographic and clinical characteristics of these patients are detailed in Table S1.

### Baseline Characteristics of the Matched Cohorts

After adjustment for baseline confounders through the PSM method, 294 patient pairs with rBCIS-JS scores ≤4 or greater were successfully matched. All standardized mean differences for the baseline confounders considered in the PSM were below 10%. Baseline characteristics of the PSM cohorts of patients are reported in Table 1. The median age was 83.0 years, and the average of Society of Thoracic Surgeons mortality risk was 5.0%. The incidences of angina, evaluated as Canadian Cardiovascular Society >1 (30.5% versus 29.0%), and reduced left ventricular ejection fraction (<40%; 20.4% versus 19.7%) were higher in patients with more myocardium jeopardized (rBCIS-JS > 4). Instead, New York Heart Association class >2 was more frequent in patients with rBCIS-JS ≤4 (64.3% versus 63.6%).

**Table 1.**
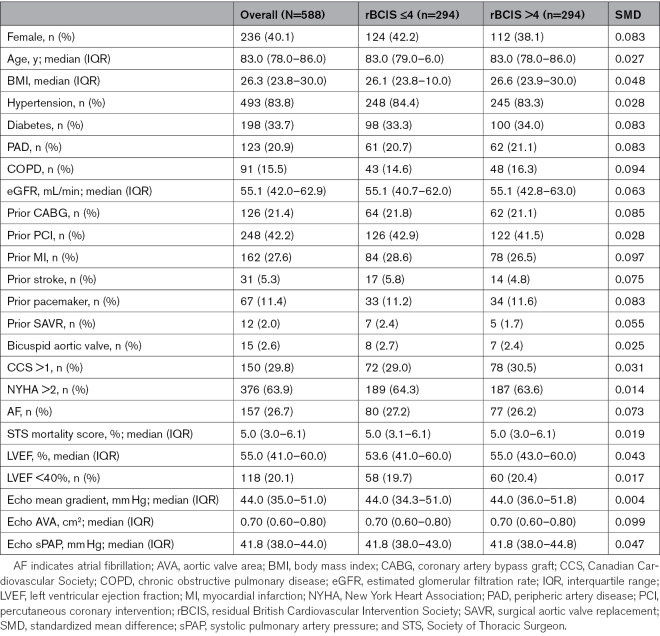
Baseline Characteristics of the Matched Population

### CAD Characteristics of the Matched Cohorts

CAD characteristics of the PSM cohorts of patients are reported in Table 2. In patients with rBCIS-JS >4, the incidence of multivessel disease (79.3% versus 42.5%, *P*<0.001), the involvement of proximal coronary segments (88.1% versus 55.4%, *P*<0.001), and the left main-proximal and left anterior descending disease (71.8% versus 24.5%, *P*<0.001) were more frequent.

**Table 2.**
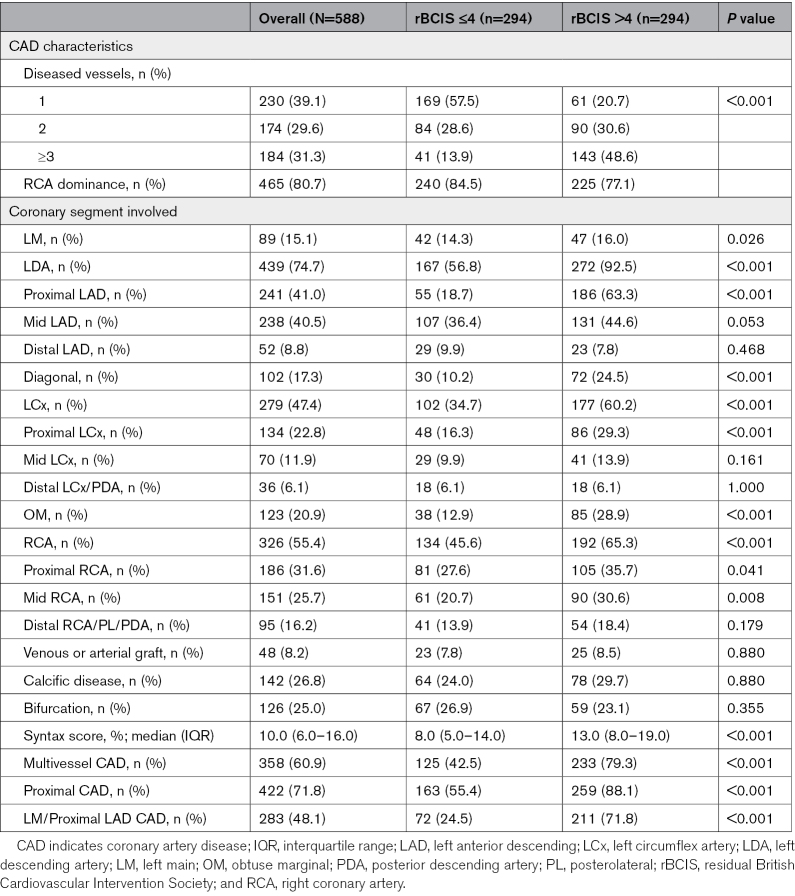
Characteristics of CAD of the Matched Population

### TAVI Procedures Characteristics of the Matched Cohorts

TAVI procedures were predominantly performed through a trans-femoral approach (n=545, 93.5%) and under local anesthesia (n=472, 80.8%). The most commonly used TAVI devices were the balloon-expandable Edwards SAPIEN 3/Ultra (Edwards LifeSciences, Irvine, CA; n=222, 37.8%) and the self-expanding Evolut R/PRO/PRO+ (Medtronic, Inc, Marlborough, MA; n=216, 36.8%). Further details of the TAVI procedures after adjustment are reported in Table 3.

**Table 3.**
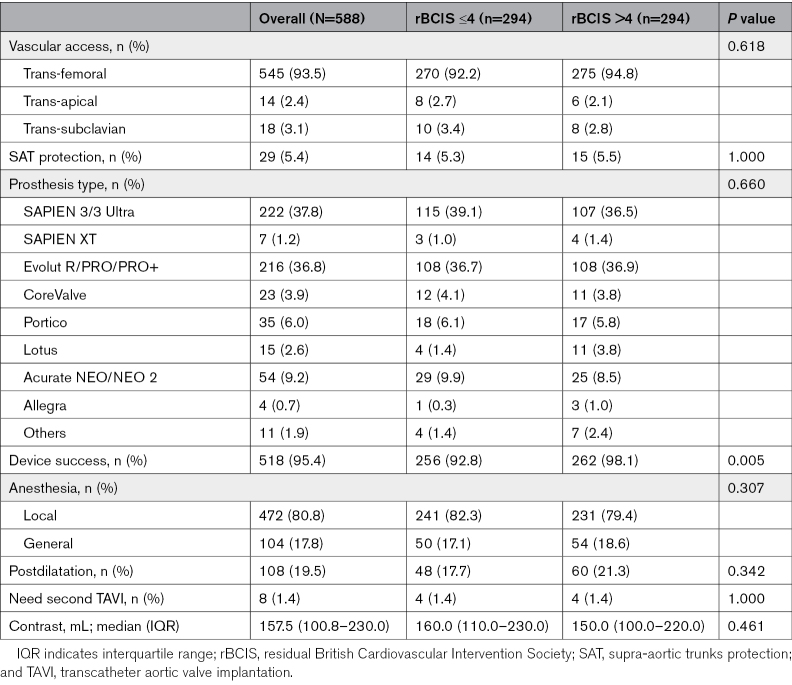
Characteristics of TAVI Procedures of the Matched Population

### Outcomes of the Matched Cohorts

**Figure 2.**
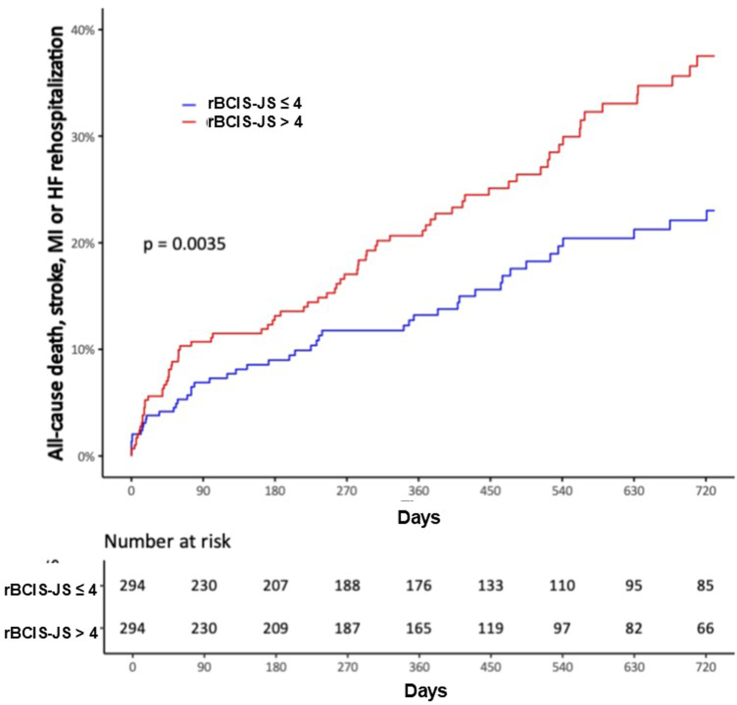
**Incidence of primary outcomes.** The figure shows the cumulative incidence of primary outcome (all-cause death, stroke, myocardial infarction [MI], and heart failure [HF] rehospitalization) in matched population stratified according to jeopardized myocardium after planned percutaneous coronary intervention (PCI) using the residual British Cardiovascular Intervention Society Jeopardy Score (rBCIS-JS).

**Figure 3.**
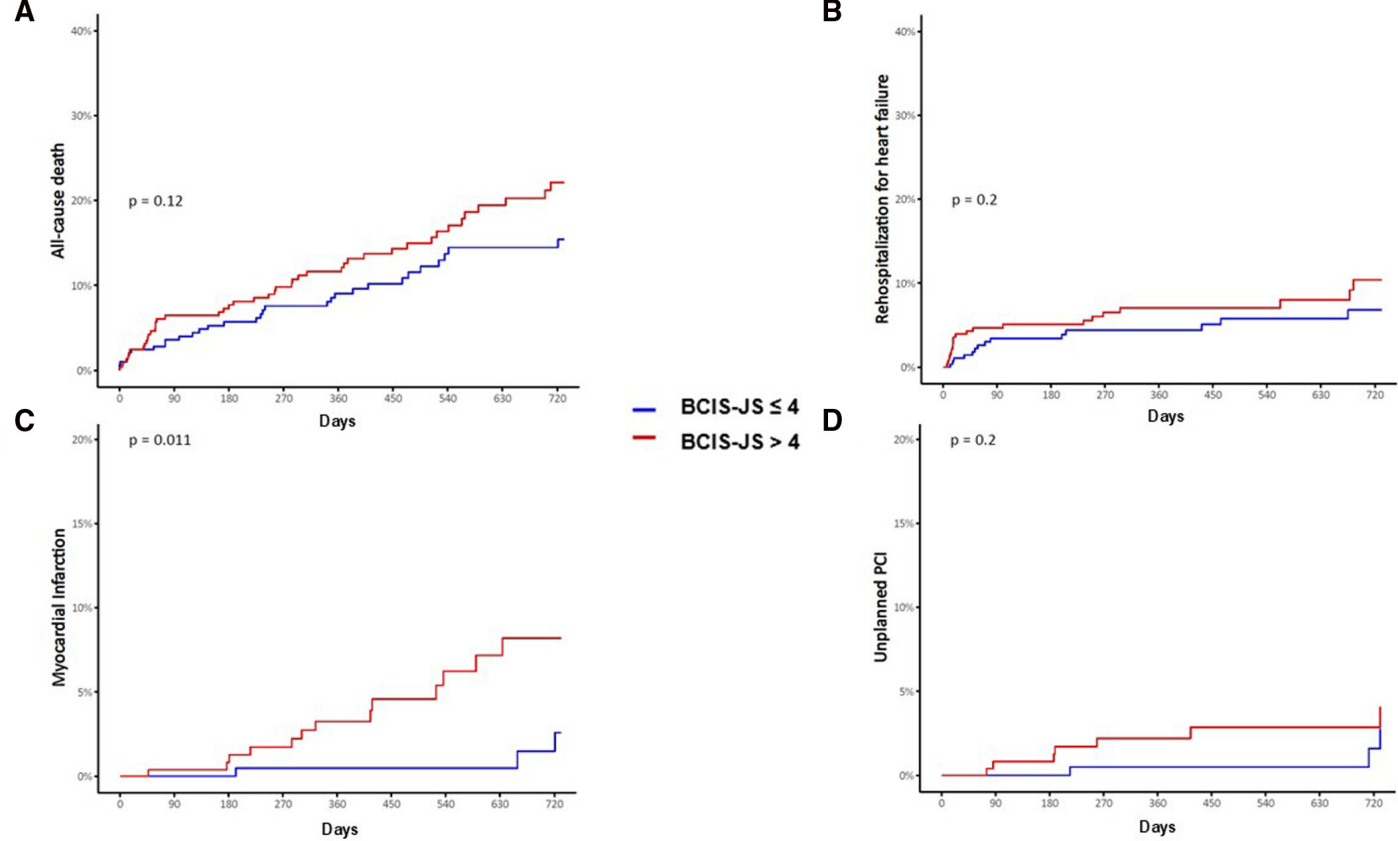
**Incidence of secondary outcomes.** The figure shows the cumulative incidence of all-cause death (**A**), heart failure rehospitalization (**B**), myocardial infarction (**C**), and unplanned percutaneous coronary intervention (PCI; **D**) in matched population stratified according to jeopardized myocardium after planned PCI using the British Cardiovascular Intervention Society Jeopardy Score (BCIS-JS).

At 2-year follow-up, the incidence of the primary end point was higher in patients with higher rBCIS-JS (37.5% versus 23.0%, *P*=0.035; Figure 2). No statistically significant differences were found in terms of all-cause death (22.2% versus 15.4%, *P*=0.12), unplanned PCI (4.1% versus 2.8%, *P*=0.2), and HF rehospitalization (10.4% versus 6.8% for, *P*=0.2) for patients with rBCIS-JS >4 or ≤4, respectively (Figure 3). Instead, the incidence of MI was more frequent in patients with higher rBCIS-JS (8.2% versus 2.6%, *P*=0.01; Figure 3). The incidence of in-hospital adverse events (death, cardiovascular death, MI, minor bleeding and major bleeding) was similar between the 2 matched groups (Table 4). In particular, the incidence of MI did not differ in the in-hospital outcome, suggesting that the difference observed at 2 years is attributable to new MI rather than periprocedural MI.

**Table 4.**
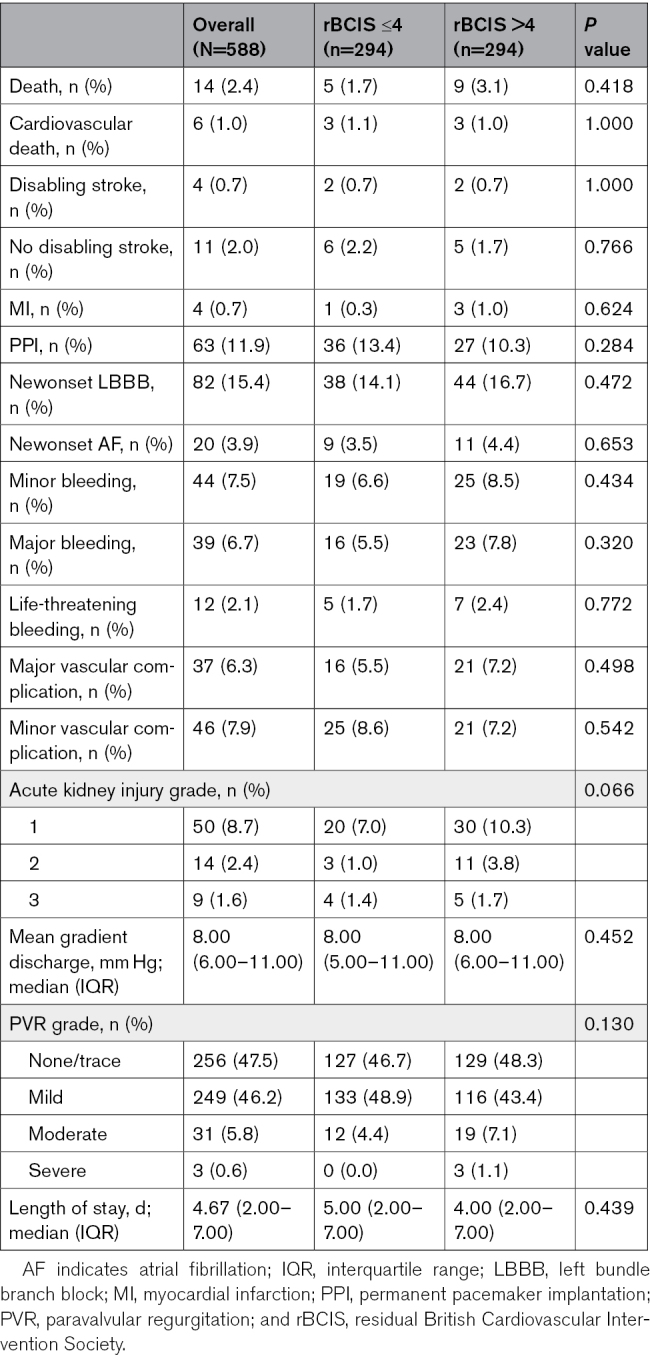
In-Hospital Outcomes of the Matched Population

### Predictors of the Primary End Point in the Overall Study Population

At multivariable Cox-regression analysis, rBCIS-JS >4 (HR, 1.43 [95% CI, 1.11–1.84]; *P*=0.005), estimated glomerular filtration rate (mL/min; HR, 0.99 [95% CI, 0.98–0.99]; *P*<0.001), atrial fibrillation (HR, 1.46 [95% CI, 1.18–1.81]; *P*<0.001), and Society of Thoracic Surgeons mortality risk score >4% (HR, 1.39 [95% CI, 1.09–1.76]; *P*=0.007) were independently associated with the 2-year primary composite end point (Table 5).

**Table 5.**
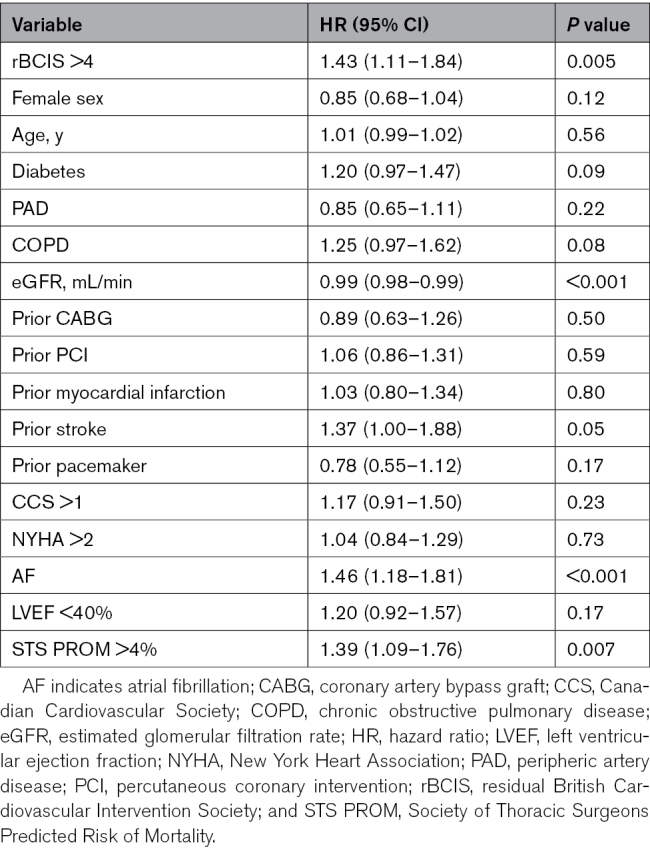
Multivariable Cox-Regression Analysis of Factors Associated With the Study Composite End Point at 2 Years

## Discussion

The optimal strategy for managing concomitant CAD in patients with severe AS who are candidates for TAVI remains debated.^3–14^ In this analysis of the international REVASC-TAVI registry, we observed that the residual extent of myocardium at risk significantly impacts clinical outcomes in patients with concomitant CAD undergoing TAVI. Specifically, coronary revascularization planning (including PCI performed before, during, and up to 30 days after TAVI) that results in a small amount of residual jeopardized myocardium (rBCIS-JS ≤4) improves prognosis after TAVI by reducing the incidence of the composite end point of all-cause mortality, stroke, MI, and HF rehospitalization at 2 years.

In the trials that led to the approval of TAVI, revascularization of significant coronary stenosis within 30 days before the procedure was mandatory.^17,18^ As a result, the safety of performing TAVI without prior revascularization was not explored in these studies. Despite recent evidence from randomized trials such as ACTIVATION and NOTION-3, recommendations regarding coronary revascularization in TAVI candidates remain relatively vague. The 2025 ESC/EACTS Valvular Heart Disease Guidelines provide a Class IIa recommendation for PCI in patients with ≥90% coronary stenosis in vessels ≥2.5 mm, and a Class IIb recommendation for ≥70% stenosis in proximal segments. These weak levels of evidence underscore that the optimal management of concomitant CAD in patients undergoing TAVI remains uncertain and requires further clarification in future studies. However, performing PCI in patients with AS can be challenging due to the perceived high procedural risk.^19^ Therefore, careful patient selection and strategic coronary revascularization planning are essential to achieve favorable PCI outcomes in this population.^20–24^ Notably, in addition to clinical factors, the extent of myocardium at risk and the choice of target lesions significantly influence PCI effectiveness.^10–25^ Previous studies have demonstrated that CAD severity, as measured by the Syntax Score, and residual incomplete revascularization, as evaluated by the residual Syntax Score, significantly impact mortality after TAVI.^25^ In the present study, we evaluated the extent of myocardium at risk using the BCIS-JS because, unlike other angiographic scoring systems that focus on lesion-specific characteristics (such as the Syntax Score), the BCIS-JS provides a simple classification of CAD based on the myocardial territory at risk. Similarly, our findings demonstrated that minimizing the myocardium jeopardized by CAD in patients with TAVI improves clinical outcomes at the 2-year follow-up. A previous smaller study^10^ also showed that a BCIS-JS of ≤4 before TAVI was linked to better clinical outcomes in patients with coexisting CAD and severe AS, similar to patients without concomitant CAD. All together, these evidences strongly suggest that, while not all coronary stenoses require revascularization for clinical benefit, a minimalistic approach to revascularization may be insufficient. In this regard, the evaluation of the extent of residual myocardium left unrevascularized emerges as a pivotal factor to be evaluated in the individual patient with concomitant CAD and AS. Interestingly, the REVASC-TAVI study indicated that complete myocardial revascularization, achieved either staged or concomitant with TAVI, was comparable to incomplete revascularization strategies in reducing the risk of all-cause mortality, stroke, MI, and rehospitalization for HF at 2 years.^11^ Similarly, the ACTIVATION trial showed that a routine revascularization strategy did not result in a clinical benefit for TAVI patients affected by CAD compared with a conservative management strategy.^13^

Recently, the NOTION-3 trial compared conservative management with routine PCI revascularization in patients with stable CAD and severe symptomatic aortic stenosis. The PCI approach, guided by FFR or significant coronary artery stenosis, showed a lower risk of adverse events, including death, MI, or urgent revascularization, over a 2-year follow-up period.^14^ Unlike the ACTIVATION trial, which defined CAD anatomically as a stenosis ≥70%, the NOTION-3 trial defined CAD using FFR or an angiographic stenosis ≥90%, thereby identifying the actual myocardial area at risk. Recently, the FAITAVI trial, presented at EuroPCR 2025, demonstrated that a physiology-guided PCI strategy using FFR in patients with intermediate coronary lesions undergoing TAVI was associated with improved outcomes compared with an angiography-guided approach. Therefore, both the extent of myocardium at risk and functional assessment of coronary lesions should play a central role in guiding revascularization decisions, rather than relying solely on angiographic criteria.

The occurrence of unplanned PCI after TAVI was low in patients without CAD at the time of the procedure but increased over time in patients with coexisting CAD, especially those with multivessel disease, driven mainly by acute coronary syndromes.^26^ Consistent with this, our study found an increased incidence of MI in patients with higher residual jeopardized myocardium.

In the decision-making process regarding coronary revascularization in patients with aortic stenosis and concomitant CAD, many factors should be considered, such as the extent of myocardium at risk and the clinical presentation. Our study demonstrated that minimizing the myocardium jeopardized by CAD in patients with TAVI should be prioritized to improve patient outcomes. Previous studies have demonstrated that clinical presentation with acute coronary syndrome was significantly associated with an increased incidence of cardiovascular death.^27^ Therefore, a Heart Team approach can lead the revascularization planning, reinforcing the importance of individualized decision-making rather than a one-size-fits-all strategy. This aligns with emerging evidence emphasizing that the extent of myocardium at risk, rather than the mere presence of coronary stenosis, should drive revascularization decisions.

### Limitations

This observational study lacked independent event adjudication and core laboratory imaging analysis, which may have introduced variability in event assessment across centers. Although PSM helped align baseline characteristics between the groups, the nonrandomized design implies that some unmeasured confounders may still have influenced the results. Furthermore, although PSM reduced the sample size potentially limiting statistical precision, this approach ensured excellent covariate balance and minimized confounding. In addition, the inclusion of patients with both preexisting and newly diagnosed CAD may have introduced further bias. Patients with incomplete data were excluded a priori, which may have introduced selection bias, although their demographic profile was comparable to that of the included population. CAD was defined by mixed criteria (visual angiography, FFR, instantaneous wave-free ratio, or intravascular ultrasound) across centers. Functional or imaging assessment was infrequent (7.5% and 5.5% of cases, respectively), so most lesions were identified angiographically. Despite this datum reflected guidelines recommendations during study period, this heterogeneity may have led to misclassification of CAD severity. Due to the limited number of cases assessed by physiology or intravascular imaging, sensitivity analyses stratified by diagnostic modality were not feasible. It should also be acknowledged that both angiographic assessment based on a ≥70% diameter stenosis cutoff and the functional evaluation in patients with severe aortic stenosis may overestimate the functional significance of coronary lesions. Decision to perform coronary revascularization and the choice of physiological assessment (instantaneous wave-free ratio or FFR) were left to the operator’s discretion, introducing potential selection bias and heterogeneity in management. While this real-world, operator-driven approach reflects contemporary clinical practice, it may have influenced the observed outcomes and should be interpreted accordingly. In contrast, the recent NOTION-3 and FAITAVI trials demonstrated the prognostic benefit of physiology-guided coronary revascularization, underscoring the need for more standardized approaches in future studies. We acknowledge that the definitions of planned, staged, and concomitant PCI were based on clinical intent and may therefore be somewhat subjective. Although prespecified criteria were applied for consistency, some overlap between planned staged and unplanned procedures cannot be completely excluded. The rBCIS-JS reflects the extent of myocardium at risk but does not incorporate information on the angiographic severity or plaque burden of individual lesions, which have been shown to influence outcomes, as highlighted by the NOTION-3 trial. Although the rBCIS-JS is a continuous variable, in the present study we applied the established >4 threshold, as previously validated, to allow consistency and comparability with prior research. Future analyses considering the rBCIS-JS as a continuous measure might provide additional insights into the graded relationship between residual myocardial risk and outcomes. Given the nonrandomized, observational design of this study, our findings should be considered hypothesis-generating and warrant confirmation in prospective, randomized trials.

### Conclusions

The management of concomitant CAD leading to small residual jeopardized myocardium (rBCIS-JS ≤4) improved prognosis in patients with TAVI by reducing the incidence of MI at 2-year follow-up. Minimizing the myocardium jeopardized by CAD in patients with TAVI should be pursued to improve patient outcomes. Prospective, randomized trials with longer follow-up are needed to corroborate the results of the present analysis.

## ARTICLE INFORMATION

### Disclosures

Dr Burzotta discloses having received speaker’s fees from Abbott, Abiomed, Medtronic, and Edwards Lifesciences. Dr Aurigemma discloses having received speaker’s fees from Abbott, Abiomed, Medtronic, Edwards Lifesciences, and Daiichi-Sankyo. Dr Barbanti discloses being a consultant for Edwards Lifesciences, Medtronic, and Boston Scientific. Dr Pilgrim reports research grants from the Swiss National Science Foundation, the Swiss Heart Foundation, the Swiss Polar Institute, and the Bangerter-Rhyner Foundation; research, travel, or educational grants to the institution without personal remuneration from Biotronik, Boston Scientific, Edwards Lifesciences, and ATSens; and speaker and consultancy fees to the institution from Biotronik, Boston Scientific, Edwards Lifesciences, Abbott, Medtronic, Biosensors, and Highlife. Dr Andreas discloses being a proctor/consultant/speaker for Edwards, Abbott, Medtronic, Boston Scientific, BBraun, and Zoll, and receiving institutional research grants from Edwards, Abbott, Medtronic, and Lifesciences International. Dr De Backer discloses receiving institutional research grants and consulting fees from Abbott, Boston Scientific, and Medtronic. Dr Bedogni discloses being a consultant and proctor for Abbott, Medtronic, Meril, and Boston Scientific Corporation. Dr Bunc discloses having received speaker’s fee from Prictor Abbott, Meril, Edwards, and Medtronic. Dr Tamarasso being a consultant or receiving consultancy fees from Abbott, Edwards Lifesciences, Medtronic, Shenqi Medical, Boston Scientific, CoreMedic, Picardia, Cardiovalve, CoreQuest, and Startric. Dr Palmerini discloses receiving lecture fees from Edwards and Medtronic and being a proctor Medtronic. Dr Gautier discloses being a consultant for Boston Scientific. Dr Kim discloses receiving personal fees from Abbott, Boston Scientific, Edwards Lifesciences, JenaValve, Meril Life Sciences, HID Imaging, and P&F; institutional fees from Boston Scientific. Dr Savontaus discloses being a proctor for Medtronic and Boston Scientific. Dr Wienemann reports travel grants from JenaValve Technology. Dr Watanabe discloses being a consultant and proctor for Edwards, Abbott, and Medtronic. Dr Porto reports consultant or speaker fees (minor) from Medtronic, Edwards, Siemens, GE, Abiomed, Philips, Sanofi, Amgen, Daiichi-Sankyo, AstraZeneca, Bayer, and PIAM Pharma, not related to this work. Dr Saia discloses being on advisory boards and receiving speaker’s fees from Edwards, Medtronic, Abbott, Boston Scientific, Amgen, Amarin, Bayer, Sanofi, Shockwave, and Philips. The other authors report no conflicts.

### Supplemental Material

Table S1

Figure S1

## Supplementary Material

## References

[1] Otto CM Burwash IG Legget ME Munt BI Fujioka M Healy NL Kraft KD Miyake-Hull CY Schwaegler RG. Prospective study of asymptomatic valvular aortic stenosis: clinical, echocardiographic, and exercise predictors of outcome. Circulation. 1997;95:2262–2270. doi: 10.1161/01.cir.95.9.2262 9142003 10.1161/01.cir.95.9.2262

[2] Rapp AH Hillis LD Lange RA Cigarroa JE. Prevalence of coronary artery disease in patients with aortic stenosis with and without angina pectoris. Am J Cardiol. 2001;87:1216–7; A7. doi: 10.1016/s0002-9149(01)01501-6 11356405 10.1016/s0002-9149(01)01501-6

[3] Beckmann A Hamm C Figulla HR Cremer J Kuck KH Lange R Zahn R Sack S Schuler GC Walther T; GARY Executive Board. The German Aortic Valve Registry (GARY): a nationwide registry for patients undergoing invasive therapy for severe aortic valve stenosis. Thorac Cardiovasc Surg. 2012;60:319–325. doi: 10.1055/s-0032-1323155 22859310 10.1055/s-0032-1323155

[4] Eltchaninoff H Prat A Gilard M Leguerrier A Blanchard D Fournial G Iung B Donzeau-Gouge P Tribouilloy C Debrux JL; FRANCE Registry Investigators. Transcatheter aortic valve implantation: early results of the FRANCE (French Aortic National CoreValve and Edwards) registry. Eur Heart J. 2011;32:191–197. doi: 10.1093/eurheartj/ehq261 20843959 10.1093/eurheartj/ehq261

[5] Smith CR Leon MB Mack MJ Miller DC Moses JW Svensson LG Tuzcu EM Webb JG Fontana GP Makkar RR; PARTNER Trial Investigators. Transcatheter versus surgical aortic-valve replacement in high-risk patients. N Engl J Med. 2011;364:2187–2198. doi: 10.1056/NEJMoa1103510 21639811 10.1056/NEJMoa1103510

[6] Malmberg M Gunn J Sipilä J Pikkarainen E Rautava P Kytö V. Comparison of long-term outcomes of patients having surgical aortic valve replacement with versus without simultaneous coronary artery bypass grafting. Am J Cardiol. 2020;125:964–969. doi: 10.1016/j.amjcard.2019.12.015 31948663 10.1016/j.amjcard.2019.12.015

[7] Barbanti M Buccheri S Capodanno D D’Errigo P Ranucci M Rosato S Santoro G Fusco D Tamburino C Biancari F; OBSERVANT Research Group. Transcatheter or surgical treatment of severe aortic stenosis and coronary artery disease: a comparative analysis from the Italian OBSERVANT study. Int J Cardiol. 2018;270:102–106. doi: 10.1016/j.ijcard.2018.06.011 29903519 10.1016/j.ijcard.2018.06.011

[8] Witberg G Zusman O Codner P Assali A Kornowski R. Impact of coronary artery revascularization completeness on outcomes of patients with coronary artery disease undergoing transcatheter aortic valve replacement: a meta-analysis of studies using the residual SYNTAX score (synergy between PCI with Taxus and cardiac surgery). Circ Cardiovasc Interv. 2018;11:e006000. doi: 10.1161/CIRCINTERVENTIONS.117.006000 29870384 10.1161/CIRCINTERVENTIONS.117.006000

[9] Aurigemma C Giannico MB Burzotta F Romagnoli E Cangemi S Bianchini F Bruno P Leone AM Gaspardone A Crea F. Clinical impact of the extent of jeopardized myocardium in patients undergoing transcatheter aortic valve intervention. Rev Esp Cardiol (Engl Ed). 2023;76:157–164. doi: 10.1016/j.rec.2022.05.020 35691553 10.1016/j.rec.2022.05.020

[10] Costa G Pilgrim T Amat Santos IJ De Backer O Kim WK Barbosa Ribeiro H Saia F Bunc M Tchetche D Garot P; REVASC-TAVI Registry. Management of myocardial revascularization in patients with stable coronary artery disease undergoing transcatheter aortic valve implantation. Circ Cardiovasc Interv. 2022;15:e012417. doi: 10.1161/CIRCINTERVENTIONS.122.012417 36538579 10.1161/CIRCINTERVENTIONS.122.012417

[11] Aurigemma C Massussi M Fraccaro C Adamo M D’Errigo P Rosato S Seccareccia F Santoro G Baiocchi M Barbanti M; OBSERVANT II Research Group. Impact of chronic coronary artery disease and revascularization strategy in patients with severe aortic stenosis who underwent transcatheter aortic valve implantation. Am J Cardiol. 2023;206:14–22. doi: 10.1016/j.amjcard.2023.08.045 37677878 10.1016/j.amjcard.2023.08.045

[12] Patterson T Clayton T Dodd M Khawaja Z Morice MC Wilson K Kim WK Meneveau N Hambrecht R Byrne J; ACTIVATION Trial Investigators. ACTIVATION (Percutaneous Coronary Intervention Prior to Transcatheter Aortic Valve Implantation): a randomized clinical trial. JACC Cardiovasc Interv. 2021;14:1965–1974. doi: 10.1016/j.jcin.2021.06.041 34556269 10.1016/j.jcin.2021.06.041

[13] Lønborg J Jabbari R Sabbah M Veien KT Niemelä M Freeman P Linder R Ioanes D Terkelsen CJ Kajander OA; NOTION-3 Study Group. PCI in patients undergoing transcatheter aortic-valve implantation. N Engl J Med. 2024;391:2189–2200. doi: 10.1056/nejmoa2401513 39216095 10.1056/NEJMoa2401513

[14] Praz F Borger MA Lanz J Marin-Cuartas M Abreu A Adamo M Ajmone Marsan N Barili F Bonaros N Cosyns B; ESC/EACTS Scientific Document Group. 2025 ESC/EACTS guidelines for the management of valvular heart disease. Eur Heart J. 2025;46:4635–4736. doi: 10.1093/eurheartj/ehaf194 40878295 10.1093/eurheartj/ehaf194

[15] De Silva K Morton G Sicard P Chong E Indermuehle A Clapp B Thomas M Redwood S Perera D. Prognostic utility of BCIS myocardial jeopardy score for classification of coronary disease burden and completeness of revascularization. Am J Cardiol. 2013;111:172–177. doi: 10.1016/j.amjcard.2012.09.012 23102883 10.1016/j.amjcard.2012.09.012

[16] Kappetein AP Head SJ Généreux P Piazza N van Mieghem NM Blackstone EH Brott TG Cohen DJ Cutlip DE van Es GA; Valve Academic Research Consortium-2. Updated standardized endpoint definitions for transcatheter aortic valve implantation: the Valve Academic Research Consortium-2 consensus document. J Thorac Cardiovasc Surg. 2013;145:6–23. doi: 10.1016/j.jtcvs.2012.09.002 23084102 10.1016/j.jtcvs.2012.09.002

[17] Kodali SK Williams MR Smith CR Svensson LG Webb JG Makkar RR Fontana GP Dewey TM Thourani VH Pichard AD; PARTNER Trial Investigators. Two-year outcomes after transcatheter or surgical aortic-valve replacement. N Engl J Med. 2012;366:1686–1695. doi: 10.1056/NEJMoa1200384 22443479 10.1056/NEJMoa1200384

[18] Adams DH Popma JJ Reardon MJ Yakubov SJ Coselli JS Deeb GM Gleason TG Buchbinder M Hermiller J Jr Kleiman NS; U.S. CoreValve Clinical Investigators. Transcatheter aortic-valve replacement with a self-expanding prosthesis. N Engl J Med. 2014;370:1790–1798. doi: 10.1056/NEJMoa1400590 24678937 10.1056/NEJMoa1400590

[19] Paradis JM Fried J Nazif T Kirtane A Harjai K Khalique O Grubb K George I Hahn R Williams M. Aortic stenosis and coronary artery disease: what do we know? What don’t we know? A comprehensive review of the literature with proposed treatment algorithms. Eur Heart J. 2014;35:2069–2082. doi: 10.1093/eurheartj/ehu247 24970334 10.1093/eurheartj/ehu247

[20] Goel SS Agarwal S Tuzcu EM Ellis SG Svensson LG Zaman T Bajaj N Joseph L Patel NS Aksoy O. Percutaneous coronary intervention in patients with severe aortic stenosis. Implication for transcatheter aortic valve replacement. Circulation. 2012;125:1005–1013. doi: 10.1161/CIRCULATIONAHA.111.039180 22282327 10.1161/CIRCULATIONAHA.111.039180

[21] Gasparetto V Fraccaro C Tarantini G Buja P D’Onofrio A Yzeiraj E Pittarello D Isabella G Gerosa G Iliceto S. Safety and effectiveness of a selective strategy for coronary artery revascularization before transcatheter aortic valve implantation. Catheter Cardiovasc Interv. 2013;81:376–383. doi: 10.1002/ccd.24434 22461314 10.1002/ccd.24434

[22] Abdel-Wahab M Mostafa AE Geist V Stöcker B Gordian K Merten C Richardt D Toelg R Richardt G. Comparison of outcomes in patients having isolated transcatheter aortic valve implantation versus combined with preprocedural percutaneous coronary intervention. Am J Cardiol. 2012;109:581–586. doi: 10.1016/j.amjcard.2011.09.053 22133754 10.1016/j.amjcard.2011.09.053

[23] Wendt D Kahlert P Lenze T Neuhäuser M Price V Konorza T Erbel R Jakob H Thielmann M. Management of high-risk patients with aortic stenosis and coronary artery disease. Ann Thorac Surg. 2013;95:599–605. doi: 10.1016/j.athoracsur.2012.07.075 23021302 10.1016/j.athoracsur.2012.07.075

[24] Chakravarty T Sharma R Abramowitz Y Kapadia S Latib A Jilaihawi H Poddar KL Giustino G Ribeiro HB Tchetche D. Outcomes in patients with transcatheter aortic valve replacement and left main stenting: the TAVR-LM registry. J Am Coll Cardiol. 2016;67:951–960. doi: 10.1016/j.jacc.2015.10.103 26916485 10.1016/j.jacc.2015.10.103 PMC5091082

[25] D’Ascenzo F Verardi R Visconti M Conrotto F Scacciatella P Dziewierz A Stefanini GG Paradis JM Omedè P Kodali S. Independent impact of extent of coronary artery disease and percutaneous revascularisation on 30-day and one-year mortality after TAVI: a meta-analysis of adjusted observational results. EuroIntervention. 2018;14:e1169–e1177. doi: 10.4244/EIJ-D-18-00098 30082258 10.4244/EIJ-D-18-00098

[26] Okuno T Demirel C Tomii D Heg D Häner J Siontis GCM Lanz J Räber L Strotecky S Fürholz M. Long-term risk of unplanned percutaneous coronary intervention after transcatheter aortic valve replacement. EuroIntervention. 2022;18:797–803. doi: 10.4244/EIJ-D-22-00342 36039573 10.4244/EIJ-D-22-00342 PMC9725053

[27] Saia F Palmerini T Compagnone M Battistini P Moretti C Taglieri N Marcelli C Bruno AG Ghetti G Corsini A. Coronary artery disease and reasonably incomplete coronary revascularization in high-risk patients undergoing transcatheter aortic valve implantation. Catheter Cardiovasc Interv. 2020;95:19–27. doi: 10.1002/ccd.28211 30916884 10.1002/ccd.28211

