# Supplementary material for: Clinical Impact of Myocardium at Risk in Transcatheter Aortic Valve Implantation
Source: Circ Cardiovasc Interv. 2026 May 20;19(7):e015770. doi: 10.1161/CIRCINTERVENTIONS.125.015770 (PMC13384366; doi:10.1161/CIRCINTERVENTIONS.125.015770)
Supplement: Supplementary file 1 [file hcv-19-e015770-s001.pdf]

SUPPLEMENTAL MATERIAL

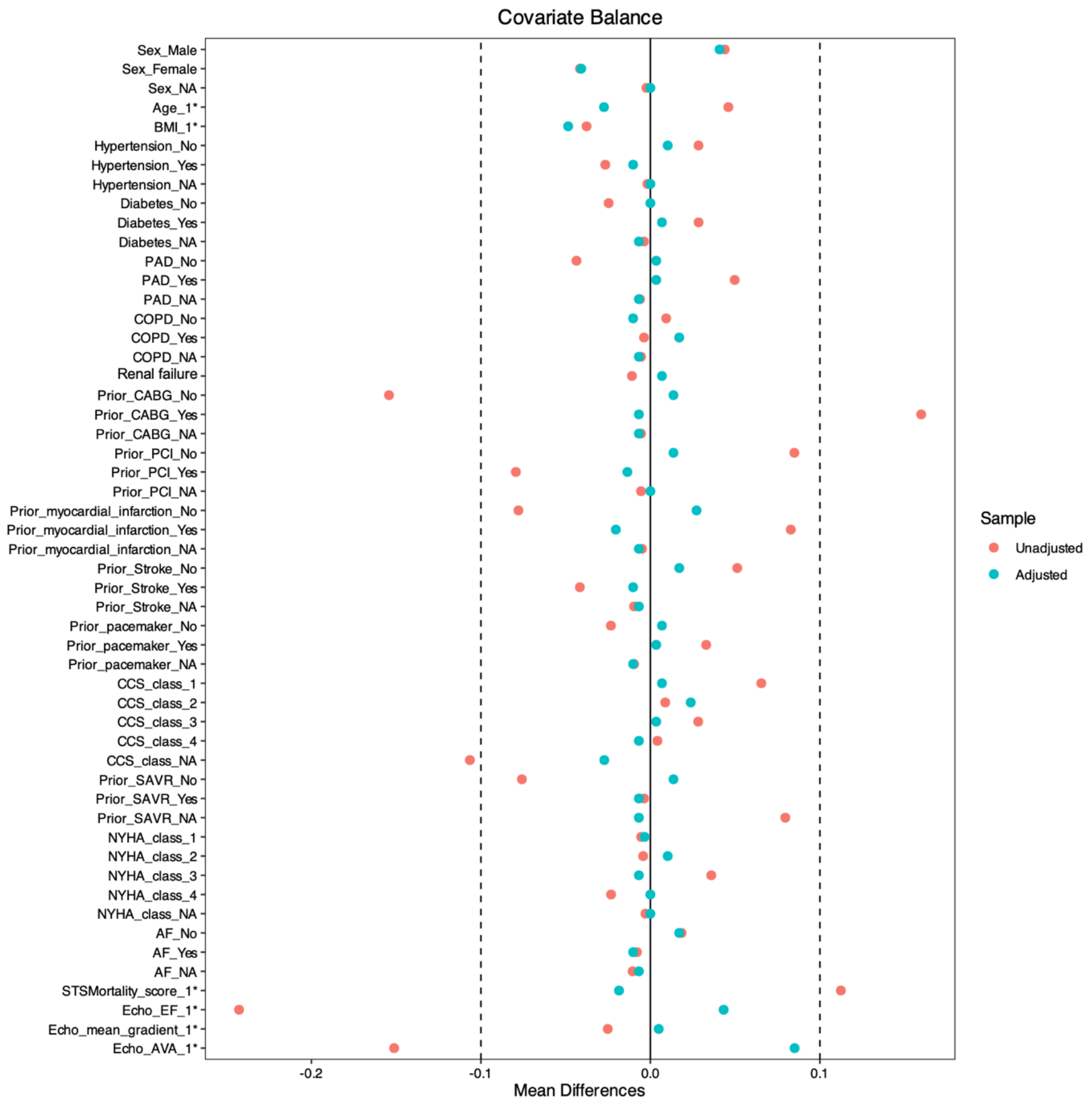

Covariate Balance
Sex_Male
Sex_Female
Sex_NA
Age_1*
BMI_1*
Hypertension_No
Hypertension_Yes
Hypertension_NA
Diabetes_No
Diabetes_Yes
Diabetes_NA
PAD_No
PAD_Yes
PAD_NA
COPD_No
COPD_Yes
COPD_NA
Renal failure
Prior_CABG_No
Prior_CABG_Yes
Prior_CABG_NA
Prior_PCI_No
Prior_PCI_Yes
Prior_PCI_NA
Prior_myocardial_infarction_No
Prior_myocardial_infarction_Yes
Prior_myocardial_infarction_NA
Prior_Stroke_No
Prior_Stroke_Yes
Prior_Stroke_NA
Prior_pacemaker_No
Prior_pacemaker_Yes
Prior_pacemaker_NA
CCS_class_1
CCS_class_2
CCS_class_3
CCS_class_4
CCS_class_NA
Prior_SAVR_No
Prior_SAVR_Yes
Prior_SAVR_NA
NYHA_class_1
NYHA_class_2
NYHA_class_3
NYHA_class_4
NYHA_class_NA
AF_No
AF_Yes
AF_NA
STSMortality_score_1*
Echo_EF_1*
Echo_mean_gradient_1*
Echo_AVA_1*
-0.2
-0.1
0.0
0.1
Mean Differences
Sample
Unadjusted
Adjusted

Supplemental Table 1. Baseline Characteristics of the Unmatched Population

	**Overall** (n=2025)	**rBCIS-JS≤4** (n=1714)	**rBCIS>4** (n=311)	SMD
Female, n (%)	833 (41.1)	716 (41.8)	117 (37.6)	0.111
Age, years, median [IQR]	82.4 [78.0-85.8]	82.2 [78.0-85.4]	83.0 [78.2-86.0]	0.046
BMI, median [IQR]	26.5 [23.8-29.4]	26.6 [23.8-29.3]	26.40 [23.53-29.8]	0.038
Hypertension, n (%)	1719 (84.9)	1462 (85.3)	257 (82.6)	0.097
Diabetes, n (%)	648 (32.0)	541 (31.6)	107 (34.4)	0.079
PAD, n (%)	338 (16.7)	273 (15.9)	65 (20.9)	0.148
COPD, n (%)	319 (15.8)	271 (15.8)	48 (15.4)	0.073
eGFR, ml/min, median [IQR]	55.1 [43.0-64.5]	55.1 [43.0-65.0]	55.1 [42.9-63.0]	0.027
Prior CABG, n (%)	195 (9.6)	123 (7.2)	72 (23.2)	0.461
Prior_PCI n (%)	963 (47.6)	836 (48.8)	127 (40.8)	0.181
Prior myocardial infarction, n (%)	405 (20.0)	321 (18.7)	84 (27.0)	0.207
Prior stroke, n (%)	169 (8.3)	154 (9.0)	15 (4.8)	0.186
Prior pacemaker, n (%)	178 (8.8)	142 (8.3)	36 (11.6)	0.134
Prior SAVR, n (%)	39 (1.9)	34 (2.0)	5 (1.6)	0.293
Bicuspid aortic valve, n (%)	80 (4.0)	73 (4.3)	7 (2.3)	0.320
CCS>1, n (%)	444 (28.1)	365 (27.8)	79 (29.2)	0.029
NYHA>2, n (%)	1274 (63.1)	1075 (62.9)	199 (64.0)	0.023
AF, n (%)	541 (26.7)	460 (26.8)	81 (26.0)	0.101
STS mortality score, %, median [IQR]	5.0 [3.1-5.1]	5.0 [3.1-5.0]	5.0 [3.0-6.0]	0.121
LVEF, %, median [IQR]	55.0 [45.0-61.0]	56.0 [48.0-62.0]	55.0 [43.0-60.0]	0.250
LVEF<40%, n (%)	300 (14.8)	237 (13.8)	63 (20.3)	0.172
Echo mean gradient, mmHg, median [IQR]	44.0 [36.0-51.0]	44.0 [36.0-51.0]	44.0 [36.0-51.0]	0.023
Echo AVA, cm^2, median [IQR]	0.7 [0.6-0.8]	0.7 [0.6-0.8]	0.7 [0.6-0.8]	0.034
Echo sPAP, mmHg, median [IQR]	41.8 [37.0-41.8]	41.8 [37.0-41.8]	41.8 [38.0-43.0]	0.108
